# A Preview of Selected Articles

**DOI:** 10.1002/sctm.18-0158

**Published:** 2018-08-15

**Authors:** Stuart P. Atkinson

**Affiliations:** ^1^ Centro de Investigación Príncipe Felipe Valencia Spain

Vascular endothelial cells (ECs) line the interior walls of blood vessels where they regulate wall permeability, control blood flow, and mediate multifunctional paracrine and endocrine effects 1. The cost‐effective and efficient differentiation of ECs from stem cells may boost the development of therapeutic interventions for conditions such as cardiovascular disease (CVD) or aid in the reconstruction of damaged vessels 2. The production of ECs from pluripotent stem cells, including embryonic stem cells and patient‐specific induced pluripotent stem cells (iPSCs), may provide a means to generate sufficient cells for the said applications, although noninvasively procured autologous stem cell sources may represent the optimal source. Our first Feature Article from Liu et al. establishes the differentiation of urine‐derived stem cells (USCs) as a noninvasive, simple, and low‐cost method to generate autologous ECs for applications in advanced vascular tissue engineering strategies. In a Related Article, Cochrane et al. describe how the Quaking isoform 5 (QKI‐5) RNA‐binding protein promotes the differentiation of iPSCs into ECs in a study that may facilitate the construction of efficient patient‐specific regenerative therapies for CVD.

Microvesicles (MVs) and exosomes are two important types of extracellular vesicles (EVs): membrane‐derived phospholipid bilayer‐enclosed particles present in various biological fluids that contain microRNAs, mRNAs, proteins, and bioactive lipids 3. The secretion of EVs facilitates close‐ and far‐range cell‐to‐cell communication, and fascinating research has also begun to uncover crucial roles for stem cell‐derived EVs in tissue repair, regeneration, and immunomodulation 4. These findings have prompted considerable interest in the contents of stem cell‐derived EVs and their mechanisms of action with the aim of developing safe and efficient cell‐free therapeutic strategies for a wide range of diseases and disorders, including acute lung injury (ALI) and radiation‐induced pathologies. Our second Featured Article from Hu et al. establishes that mesenchymal stem cell‐derived MVs (MSC‐MVs) can restore lost lung function by decreasing protein permeability across lung ECs in part by increasing angiopoietin1 (Ang1) secretion and maintaining intracellular junctions. In a Related Article, Schoefinius et al. report the potential for MSC‐derived EVs (MSC‐EVs) as an effective first‐line treatment to combat radiation‐induced hematopoietic failure and a potential means to alleviate myelosuppression due to chemotherapy and toxic drug reaction.

## Featured Articles

### Promoting the Development of Vessel Tissue Engineering Strategies with USCs

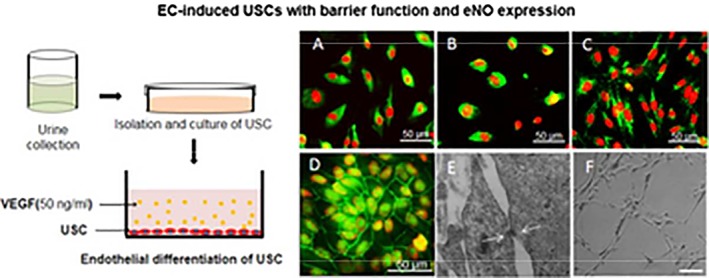

The application of tissue‐engineered vascular grafts constructed with autologous ECs in combination with suitable scaffolds may avoid many of the problems associated with revascularization approaches employing autologous vessels or allogenic/synthetic vascular grafts. As a means to generate sufficient autologous ECs, researchers from the laboratory of Yuanyuan Zhang (Wake Forest School of Medicine, Winston‐Salem, NC) recently assessed the possible generation of ECs from USCs, cells originating from the parietal cells in kidney glomeruli that possess robust proliferative potential, multipotential differentiation, and paracrine effects. Reporting in *Stem Cells Translational Medicine*, Liu et al. reveal that the directed differentiation of USCs permits the generation of cells (USC‐ECs) with endothelial morphology, ultrastructure, and functional marker expression 5. Furthermore, USC‐ECs displayed robust endothelial‐like function during in vitro analysis and expressed high levels of endothelial markers following grafting in vivo. The authors propose the production of ECs from USCs as a noninvasive, simple, and low‐cost method to generate sufficient autologous cells for applications in tissue‐engineered vascular regeneration or the repair of endothelial dysfunction.

DOI: http://10.0.3.234/sctm.18-0040

### Discovering How MSC‐MVs Repair Acute Lung Injury

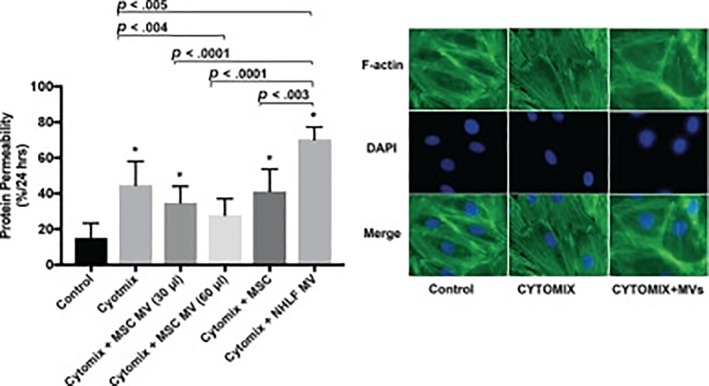

Research from the laboratory of Jae‐Woo Lee (University of California San Francisco, San Francisco, CA) previously demonstrated that MSC‐MV therapy improved many of the pathologies associated with induced ALI in mice, including the unwanted increase in protein permeability in the lung. Now, the team returns with a new *Stem Cells Transitional Medicine* article to describe the underlying mechanisms behind the therapeutic effects of MSC‐MVs by analyzing a mouse model of proinflammatory cytokine (cytomix)‐induced lung hyperpermeability 6. Overall, Hu et al. demonstrated that MSC‐MVs restore protein permeability, in part, by incorporating into inflamed human lung microvascular ECs via the CD44 receptor and increasing intracellular levels of Ang1 mRNA. The subsequent increase in Ang1 protein secretion is associated with the restoration of proper endothelial permeability via the maintenance of intercellular junction status and a reduction in the formation of actin stress fibers. As angiopoietin proteins play essential roles in vascular development and angiogenesis, and Ang1 contributes to blood vessel maturation and stability, the authors anticipate that these findings will propel the development of advanced pharmacologic therapies that will improve clinical outcomes for ALI patients.

DOI: http://10.0.3.234/sctm.17-0278

## Related Articles

### How RNA‐Binding Proteins Influence EC Differentiation and Function

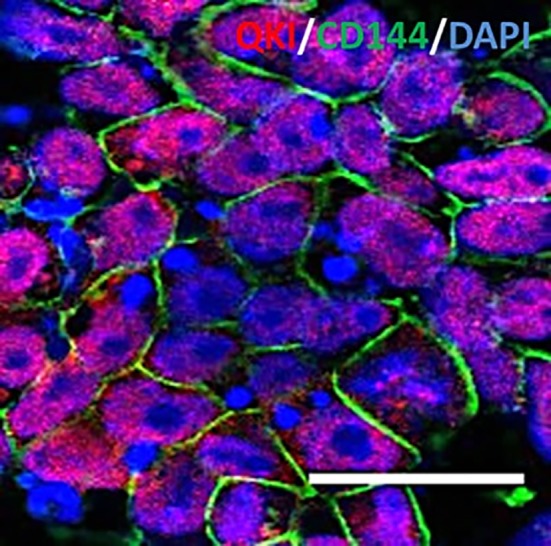

The progressive dysfunction of vascular ECs correlates with the development of CVD, one of the leading causes of mortality worldwide. As previous studies established high expression of the QKI‐5 RNA‐binding protein during directed differentiation of iPSCs into ECs, researchers led by Andriana Margariti (Queen's University Belfast, U.K.) sought to discover if modulating QKI‐5 expression could contribute to an enhanced EC differentiation strategy. In their *Stem Cells* study 7, Cochrane et al. reported that overexpression of QKI‐5 in iPSCs improves EC‐directed differentiation via QKI‐5‐mediated stabilization of STAT3 RNA and subsequent increases in VEGFR2 transcriptional activation and VEGF secretion. Excitingly, QKI‐5‐mediated improvements boosted the angiogenic behavior of iPSC‐derived ECs in Matrigel plug assays for angiogenic potential following subcutaneous injection in mice and in an experimental hindlimb ischemia model, where the authors observed enhanced angiogenesis, neovascularization, and blood flow recovery. Overall, these findings establish the modulation of RNA‐binding proteins as a potentially exciting means to improve EC production from iPSCs, boost resultant EC function, and potentially improve therapeutic interventions for CVD.

DOI: http://10.0.3.234/stem.2594

### MSC‐Derived EVs as a Therapy for Radiation‐Induced Hematopoietic Failure

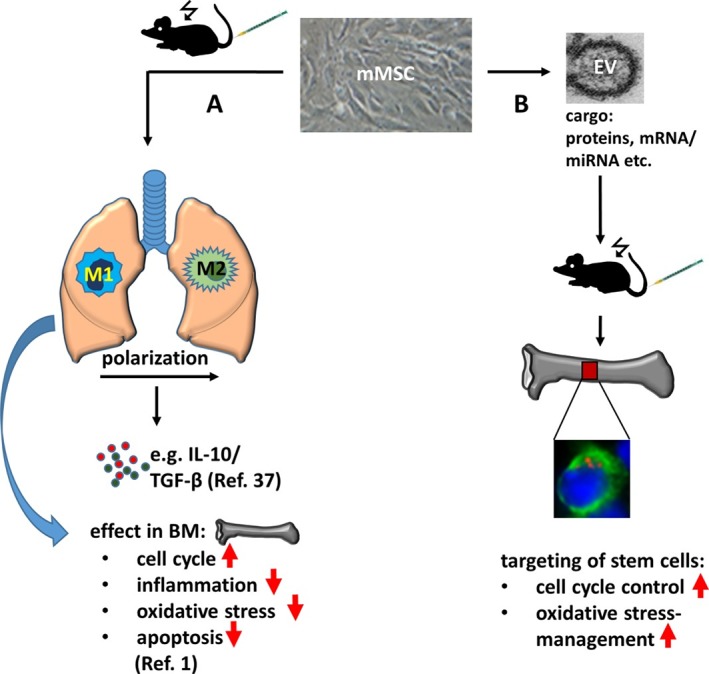

Researchers from the laboratory of Claudia Lange (University Medical Center Hamburg‐Eppendorf, Hamburg, Germany) previously demonstrated that MSC therapy boosted long‐term survival of lethally irradiated recipient mice in a study with significant relevance to patients undergoing hematopoietic stem cell (HSC) transplantation or suffering from acute radiation sickness. The team explored the therapeutic mechanisms behind MSC treatment in lethally irradiated mice in a recent *Stem Cells* study, discovering that EVs secreted from MSC provided the radioprotective effect 8. Schoefinius et al. revealed that MSC‐EVs supported HSC recovery in vitro and, following lethal irradiation of mice, systemically infused MSC‐EVs targeted and rescued hematopoietic long‐term repopulating stem cells to promote long‐term survival. While the EV cargo that specifically mediates the radioprotective effect remains unknown, the authors of the study hope that their findings will contribute to the creation of enhanced cell‐free therapies for radiation‐induced hematopoietic failure. Furthermore, the team also suggests MSC‐EVs as a safe and efficient means to alleviate myelosuppression due to chemotherapy and toxic drug reactions.

DOI: http://10.0.3.234/stem.2716
